# Effects of modified Shu-Gan-Liang-Xue decoction combined with anastrozole on osteoblastic proliferation and differentiation of MC3T3-E1 cells

**DOI:** 10.3892/mmr.2014.2962

**Published:** 2014-11-17

**Authors:** FEI ZHOU, SHUYAN HAN, NING ZHOU, WENXIAN ZHENG, PINGPING LI

**Affiliations:** Key Laboratory of Carcinogenesis and Traditional Research (Ministry of Education), Department of Integrated Traditional Chinese and Western Medicine, Peking University Cancer Hospital and Institute, Beijing 100142, P.R. China

**Keywords:** modified Shu-Gan-Liang-Xue decoction, osteoblast-like cell, alkaline phosphatase, osteocalcin, mineralization

## Abstract

Aromatase inhibitors (AIs) are widely used in the treatment of hormone-dependent breast cancer and as a result, aromatase inhibitor-associated bone loss (AIBL) has become a major concern amongst patients receiving AI treatment. Modified Shu-Gan-Liang-Xue decoction (mSGLXD), a clinical prescription, has been used for ameliorating AIBL in patients with breast cancer for decades and has achieved good clinical efficacy. However, the mechanism underlying how mSGLXD influences bone homeostasis and alleviates AIBL has remained elusive. In the present study, mSGLXD was supplemented with Rhizoma Drynariae containing phytoestrogens, and the safety of mSGLXD was evaluated. mSGLXD did not possess estrogenic activity and significantly inhibited the proliferation of estrogen receptor-positive breast cancer cell line MCF-7, which suggested that mSGLXD was safe for postmenopausal patients with breast cancer. Subsequently, the effects of mSGLXD alone or in combination with anastrozole on osteoblastic MC3T3-E1 cell proliferation and differentiation were investigated. Cell counting kit-8, reverse transcription-polymerase chain reaction and biochemical methods, such as ELISA and alizarin red S staining, were used in the present study. It was revealed that mSGLXD not only stimulated MC3T3-E1 cell proliferation, but also upregulated alkaline phosphatase and osteocalcin gene and protein expression levels. High concentrations of anastrozole (10 or 100 μmol/l) markedly inhibited MC3T3-E1 cell proliferation, but this inhibitory effect was attenuated by mSGLXD. Furthermore, mSGLXD increased MC3T3-E1 cell mineralization following β-glycerophosphate and ascorbic acid induction. Therefore, the results of the present study suggested that mSGLXD may be a promising adjuvant therapy, with high safety and efficacy, for the prevention and treatment of AIBL in patients with breast cancer who receive AI treatment.

## Introduction

Breast cancer is the most common malignancy amongst females worldwide (1). Experimental data strongly suggest that estrogens have an important role in the development and progression of hormone-dependent breast cancer (2). Approximately two thirds of postmenopausal breast cancer cases are hormone-dependent, which means that they are estrogen receptor (ER)-positive and require estrogens for tumor growth (3). Aromatase is the rate-limiting enzyme in the synthesis of estrogens from androgenic substrates (4). Aromatase inhibitors (AIs) were found to markedly suppress plasma estrogen levels, as well as intratumoral aromatase activity in postmenopausal females with breast cancer by inhibiting or inactivating the aromatase enzyme (4–7). As a result, less estrogen becomes available to stimulate the growth of hormone-dependent breast cancer cells. Large, adjuvant randomized trials have demonstrated that AIs exhibited significant improvement in disease-free survival, time to recurrence and time to distant recurrence compared with tamoxifen (8–10). For postmenopausal females with receptor-positive breast cancers, AIs have emerged as an alternative to tamoxifen due to its superior efficacy and reduced incidence of side effects, including endometrial cancer and thromboembolism (11–13).

In addition to stimulating ER-positive breast cancer cell growth, estrogens also have an important role in maintaining normal bone mass (14). Bone remodeling, which comprises bone formation by osteoblasts and bone resorption by osteoclasts, is a dynamic metabolic process that occurs throughout life (15). Estrogens are known to regulate bone homeostasis by inhibiting osteoclast activity, as well as enhancing osteoblast proliferation and osteoblast-related collagen formation (16,17). Bone mineral density (BMD) declines in females concurrently with the onset of menopause and may be accompanied by an increased risk of fracture due to the rapid decrease in serum estrogen levels (18). AI treatment markedly reduces these low circulating estrogen levels by a further 80–90% in postmenopausal patients with breast cancer (19). Therefore, AIs lead to an increase in bone loss (osteoporosis), known as aromatase inhibitor-associated bone loss (AIBL), and a higher bone fracture rate than tamoxifen (11–13). Therefore, accelerated bone loss due to long-term estrogen deprivation has become a major concern underlying the safety of AI treatment.

Clinical evidence has shown that bisphosphonates may maintain BMD and decrease fracture risk for patients with breast cancer receiving adjuvant AI therapy (20–23). However, side effects, including renal dysfunction and osteonecrosis of the jaw, have been reported following bisphosphonate treatment (24–26). Therefore, the combination of AI treatment with alternative approaches with long-term efficacy and safety profiles, including the use of Traditional Chinese Medicine, for the management of AIBL requires further analysis.

Shu-Gan-Liang-Xue decoction (SGLXD), a clinical prescription, has been used extensively for ameliorating hot flush symptoms in patients with breast cancer receiving endocrine therapy (27). SGLXD inhibited breast tumor growth in tumor-bearing nude mice (28), and none of the component herbs exhibited estrogenic activity (29). Modified Shu-Gan-Liang-Xue decoction (mSGLXD) has been used to prevent AIBL and has achieved good clinical efficacy. SGLXD was supplemented with Rhizoma Drynariae (RD; Gu-Sui-Bu in Chinese), Caulis Piperis Kadsurae (Hai-Feng-Teng in Chinese) and Caulis Trachelospermi (Luo-Shi-Teng in Chinese), and Radix Cynanchum Strati (Baiwei in Chinese) and Fructus Schisandrae (Wuweizi in Chinese) were removed, to produce mSGLXD.

RD, the dried rhizome of *Drynaria fortunei* (Kunze) J. Sm., has been known as a kidney-tonifying and anti-osteoporosis herb for the treatment of osteoporosis and bone fractures for thousands of years in China (30,31). The natural product RD, which contains phenolic compounds, was suggested to possess estrogenic activity (32) and the methanolic extract of RD was able to increase the growth of MCF-7 cells at low concentrations (33). Although SGLXD has demonstrated anti-tumor efficacy, it was necessary to test the effects of mSGLXD on breast cancer cell proliferation due to the supplementation of RD. Therefore, the effects of mSGLXD and RD alone on the proliferation of ER-positive breast cancer cell line MCF-7 were investigated and their estrogenic activities were also evaluated. To further elucidate the role of mSGLXD in alleviating AIBL, the effects of mSGLXD alone or in combination with an AI (anastrozole) on the proliferation and differentiation of osteoblastic cell lines *in vitro* were investigated using MC3T3-E1 cells, a mouse calvaria osteoblast-like cell line (34).

## Materials and methods

### Cell culture

MCF-7 (HTB-22, a human breast cancer cell line) was purchased from the American Type Culture Collection (Rockville, MD, USA) and MC3T3-E1 (3111C0001CCC000012), an osteoblast-like cell line from the C57BL/6 mouse calvaria, was obtained from the Cell Resource Center (IBMS, CAMS/PUMC, Beijing, China). The MCF-7 cell line was grown in Dulbecco’s modified Eagle’s medium (DMEM; Bioroc, Tianjin, China) and the MC3T3-E1 cell line was cultured in α-modified minimal essential medium (α-MEM) with 292 mg/ml L-glutamine, 10 mg/l ribonucleosides and 10 mg/l deoxyribonucleosides (Bioroc Pharmaceutical & Biotech Co., Ltd, Tianjin, China). Unless specified, the medium contained 10% heat-inactivated fetal bovine serum (FBS; Gibco-BRL, Invitrogen Life Technologies, Carlsbad, CA, USA), 100 U/ml penicillin and 100 μg/ml streptomycin (Solarbio Science & Technology Co., Ltd., Beijing, China). Cells were incubated at 37°C in a humidified atmosphere with 5% CO_2_. For all experiments, routine cell culture procedures were strictly followed to maintain cell density and all subcultures were used prior to passage 20.

### Preparation of drugs

The components of SGLXD and mSGLXD are exhibited in Table I. The Chinese herbs were processed into formula granules by Beijing Tcmages Pharmaceutical Co., Ltd (Beijing, China). The quality of formula granules was monitored by Fourier transform infrared spectroscopy (FTIR) (Model IRPRestige-21; Shiamdzu Corporation, Kyoto, Japan). Prior to use, the formula granules were dissolved in deionized distilled water to achieve a concentration of 1 g/ml crude drug. The solutions were sterilized by filtration through a 0.22-μm pore-sized membrane (EMD Millipore, Billerica, MA, USA) and stored at −80°C. The concentrations of mSGLXD and RD in the present study refer to the crude drug concentrations.

### Estrogenic activity of mSGLXD and RD

Estrogenic activity was evaluated using a Dual-Luciferase^®^ reporter assay (Promega Corp., Beijing, China) based bioluminescent measurement method. The p(estrogen-responsive element)-TK-Luciferase and p(*Renilla* luciferase*)*-TK plasmids were provided by Professor Wen-Ling Han (Center for Human Disease Genomics, Peking University, Peking, China). Following transfection for 24 h, MCF-7 cells were treated with various concentrations of mSGLXD (0.625, 2.5 or 10 mg/ml), 17β-estradiol (E_2_; 10 nmol/ml; Sigma-Aldrich, St Louis, MO, USA) or RD (10 mg/ml) for 48 h, and the control group was treated with equal drug dissolved solute only, prior to being lysed for the measurement of luciferase activity. The Dual-Luciferase^®^ reporter assay system contains Passive Lysis Buffer (PLB), which can directly lyse cells. Briefly, growth media was removed from the cultured cells, which were then washed with 1X phosphate-buffered saline. Following washing 100 μl 1X PLB was added to each well and the culture plates were gently agitated for 15 min at room temperature. The lysates were then transferred to tubes and centrifuged at 12,000 × g for 10 min at 4°C. Luciferase activity was detected using chemiluminescence apparatus (Model LMax II; Molecular Devices, Sunnyvale, CA, USA).

### Cell proliferation assays

MCF-7 and MC3T3-E1 cells were suspended in DMEM and α-MEM culture media and plated at a density of 5.0×10^3^ cells/well in 96-well culture dishes (Costar, Cambridge, MA, USA). Following 24 h of culture, the medium was replaced with complete culture medium supplemented with various concentrations of drugs. To assess the effects of mSGLXD and RD alone on MCF-7 cell proliferation, MCF-7 cells were treated with mSGLXD (1.25–50 mg/ml) or RD (1.25–50 mg/ml). To assess the effects of mSGLXD and anastrozole alone or in combination on MC3T3-E1 cell proliferation, MC3T3-E1 cells were treated with mSGLXD (0.625–10 mg/ml), anastrozole (0.01–100 μmol/l) or mSGLXD (0.625–10 mg/ml) as well as 10 or 100 μmol/l anastrozole. Following 48 h of drug treatment, the cells were incubated with cell counting kit-8 solution (CCK-8; Dojindo Molecular Technologies, Inc., Kumamoto, Japan) for 2 h. Subsequently, the absorbance (optical density, OD) at 450 nm was measured using a microplate reader (Model 680; Bio-Rad Laboratories, Hercules, CA, USA) and cell viability was calculated according to the following formula: (OD_sample_−OD_blank_)/(OD_control_−OD_blank_)×100%.

### Reverse transcription polymerase chain reaction (PCR) analysis

For analysis of alkaline phosphatase (ALP) and osteocalcin (OCN) gene expression, MC3T3-E1 cells were treated with mSGLXD (10 mg/ml) and anastrozole (10 μmol/l), alone or in combination, for 48 h. Total RNA was extracted from cells using TRIzol reagent (Invitrogen Life Technologies). The concentration and quality of the extracted RNA were measured with a NanoDrop 2000 (Thermo Fisher Scientific, Wilmington, DE, USA). The first-strand cDNA was generated using the TransScript first-strand cDNA synthesis supermix (Transgen, Beijing, China) according to the manufacturer’s instructions. Primers designed for PCR were synthesized by Sangon Biotech Co., Ltd (Shanghai, China) and are shown in Table II. The PCR assay was performed using SYBR green qPCR supermix (Applied Biosystems Life Technologies, Foster City, CA, USA) and performed in an ABI prism 7500 sequence detection system (Applied Biosystems Life Technologies). The PCR was carried out using the following conditions: 35°C for 10 min, followed by 40 cycles of 95°C for 30 sec, and 72°C for 32 sec. The amount of mRNA for each gene was calculated using the delta-delta CT (cycle threshold) method (35), and gene expression levels were normalized to GAPDH.

### Biochemical markers

MC3T3-E1 cells were cultured in a six-well culture plate (Costar) at a density of 4×10^4^ cells/well for 24 h. Following treatment with mSGLXD (10 mg/ml) and anastrozole (10 μmol/l), alone or in combination, for 48 h, cell ALP activity was determined using an ALP Assay kit (Jiancheng, Nanjing, China) according to the manufacturer’s instructions. ALP activity was normalized to total protein, as determined by bicinchoninic acid protein assay (Thermo Fisher Scientific). The OCN content in MC3T3-E1 cells was measured using a sandwich ELISA assay kit from Beijing Ke Ying Mei Technology Co. Ltd (Beijing, China).

### Mineralization assay

Bone mineralization was determined by alizarin red S (AR-S) staining. Calcium was bound selectively to AR-S and stained dark red. MC3T3-E1 cells were cultured in differentiation medium [α-MEM supplemented with 10% FBS, 10 mmol/l β-glycerophosphate and 50 μg/ml ascorbic acid (Sigma-Aldrich)] with or without mSGLXD (10 mg/ml) and anastrozole (10 μmol/l), alone or in combination, for 21 days in six-well plates (4×10^4^ cells/well). The treated cells were subsequently stained using an AR-S cell staining kit (Genmed, Shanghai, China) according to the manufacturer’s instructions. Images of the stained matrix were observed under an inverted microscope (CKX41; Olympus Corporation, Tokyo, Japan) and captured using a digital camera (Canon, Inc., Tokyo, Japan). To quantify matrix mineralization, AR-S staining was released from the cell matrix by incubation with 10% cetylpyridinium chloride in 10 mmol/l sodium phosphate (pH 7.0; Sigma-Aldrich) for 20 min. The AR-S concentration was determined by measuring the absorbance at 562 nm (36), using a microplate reader (Model 680; Bio-Rad Laboratories).

### Statistical analysis

All experiments were repeated three to five times and values are expressed as the mean ± standard deviation. All data were analyzed using one-way analysis of variance followed by least significant difference comparison using SPSS statistical software 16.0 (SPSS, Inc., Chicago, IL, USA). P<0.05 was considered to indicate a statistically significant difference between values.

## Results

### mSGLXD does not possess estrogenic activity, whereas RD alone does

**Figure 1:**
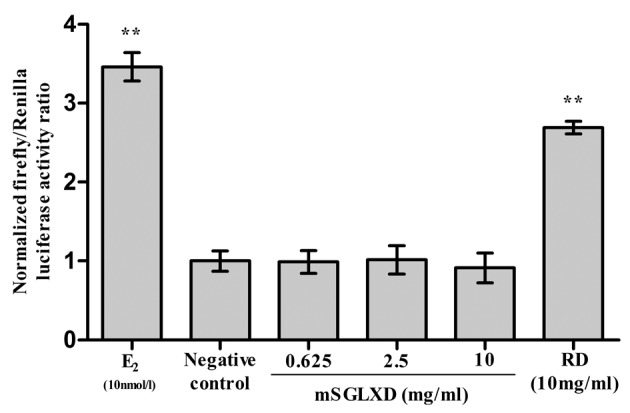
Estrogenic activity of mSGLXD and RD. Following transfection for 24 h, the culture medium was replaced by fresh medium containing E_2_ (10 nmol/l), various concentrations of mSGLXD (0.625, 2.5 or 10 mg/ml) or RD (10 mg/ml) for 48 h. The treated cells were lysed to measure luciferase activity by dual-luciferase reporter-based bioluminescent assay. Values are expressed as the mean-fold induction over negative control which was normalized to one. Values are presented as the mean ± standard deviation of three independent experiments. ^**^P<0.01 vs. negative control. mSGLXD, modified Shu-Gan-Liang-Xue decoction; RD, Rhizoma Drynariae; E_2_, 17β-estradiol.

Following treatment for 48 h, the luciferase activities in MCF-7 cells treated with various concentrations of mSGLXD were significantly lower than those induced by E_2_ (10 nmol/l; P<0.01). There was no significant difference for luciferase activities between cells treated with mSGLXD and negative control groups (P>0.05), which suggested that mSGLXD did not possess estrogenic activity (Fig. 1). The luciferase activity induced by 10 mg/ml RD was ~2.5-fold that of the negative control and mSGLXD groups (P<0.01), indicating that RD exerted estrogenic activity.

### mSGLXD inhibits MCF-7 cell proliferation

**Figure 2:**
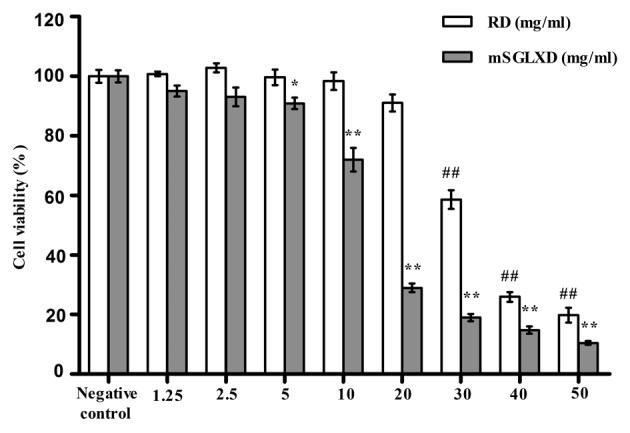
Effects of mSGLXD and RD on MCF-7 cell proliferation. Following treatment with various concentrations of mSGLXD or RD for 48 h, cell viability was measured by cell counting kit-8 analysis. Representative results from ≥three independent experiments are shown. Values are presented as the mean ± standard deviation. ^*^P<0.05, ^**^P<0.01 vs. mSGLXD negative control; ^##^P<0.01 vs. RD negative control. mSGLXD, modified Shu-Gan-Liang-Xue decoction; RD, Rhizoma Drynariae.

Following treatment for 48 h, mSGLXD significantly inhibited MCF-7 cell proliferation in a dose-dependent manner compared to that of the control group (P<0.01), while low concentrations of RD (1.25, 2.5 mg/ml) slightly promoted MCF-7 cell proliferation (P>0.05). However, RD dose-dependently inhibited MCF-7 cell proliferation in the range of 5–50 mg/ml (Fig. 2).

### mSGLXD enhances MC3T3-E1 cell proliferation and attenuates anastrozole-induced inhibition of proliferation

**Figure 3:**
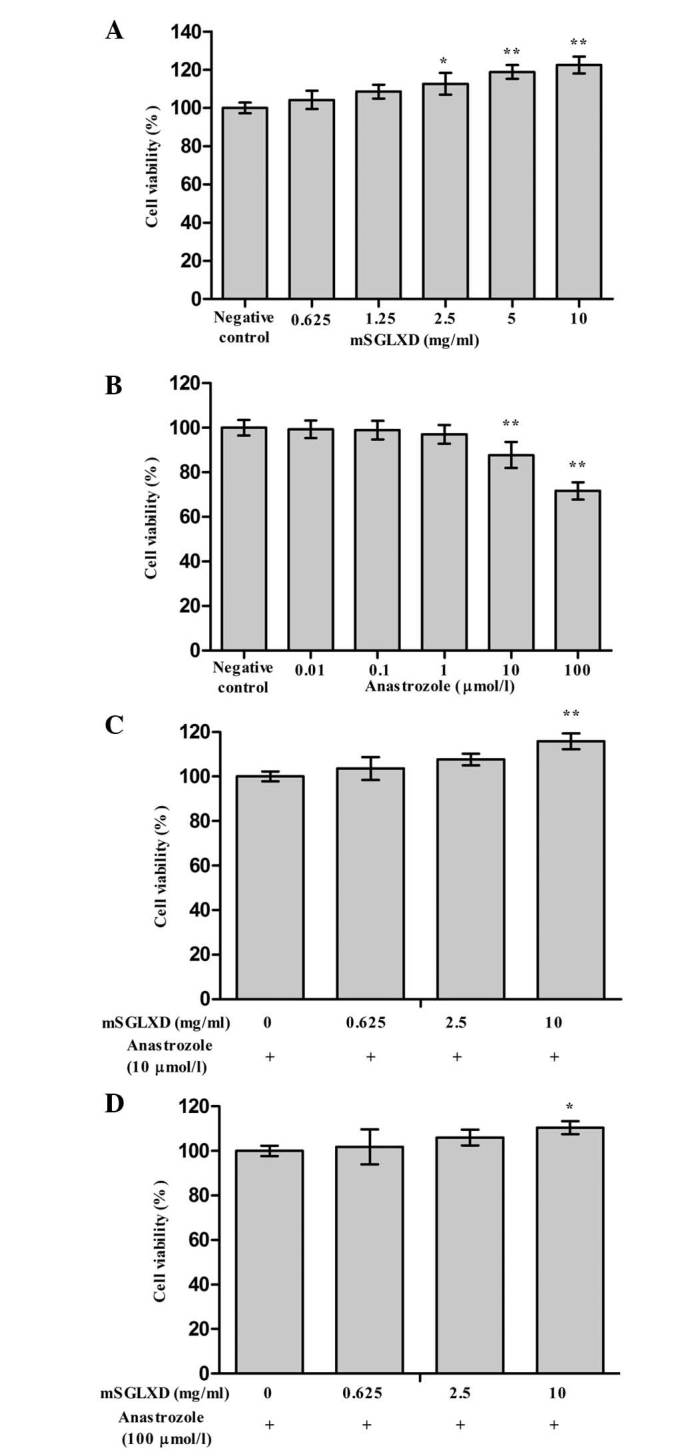
Effects of mSGLXD and anastrozole alone or in combination on MC3T3-E1 cell proliferation. MC3T3-E1 cells were treated with various concentrations of (A) mSGLXD or (B) anastrozole for 48 h, and cell viability was determined by cell counting kit-8 assay. ^*^P<0.05, ^**^P<0.01 vs. negative control, respectively). (C) Cell viability of MC3T3-E1 cells treated with various concentrations of mSGLXD combined with 10 μmol/l anastrozole for 48 h. ^**^P<0.01 vs. 10 μmol/l anastrozole alone. (D) Cell viability of MC3T3-E1 cells treated with various concentrations of mSGLXD combined with 100 μmol/l anastrozole for 48 h. ^**^P<0.01 vs. 100 μmol/l anastrozole alone. Values are presented as the mean ± standard deviation of ≥three independent experiments. mSGLXD, modified Shu-Gan-Liang-Xue decoction.

mSGLXD dose-dependently stimulated MC3T3-E1 cell proliferation in the range of 0.625–10 mg/ml following treatment for 48 h (P<0.01) (Fig. 3A). Compared with the negative control, cell viability was increased by 22.49% in the 10 mg/ml mSGLXD treatment group.

Low concentrations of anastrozole (0.01–1 μmol/l) did not influence MC3T3-E1 cell proliferation following treatment for 48 h, while high concentrations of anastrozole (10 and 100 μmol/l) inhibited MC3T3-E1 cell proliferation by 12.31 and 28.38%, respectively, compared with that of the negative control group (P<0.01; Fig. 3B).

Furthermore, mSGLXD was able to prevent 10 and 100 μmol/l anastrozole-induced MC3T3-E1 cell death, and had a more marked effect on proliferation in the 10 μmol/l anastrozole-treated group. Cells treated with combined 10 μmol/l anastrozole and 10 mg/ml mSGLXD demonstrated a significant increase in cell viability by 15.81%, as compared to cells treated with 10 μmol/l anastrozole alone (P<0.05). Lower concentrations of mSGLXD (0.625, 2.5 mg/ml) demonstrated certain protective effects against anastrozole-induced cell viability inhibition but without significant difference (Fig. 3C and D).

### mSGLXD alone or in combination with anastrozole enhances ALP and OCN mRNA expression

**Figure 4:**
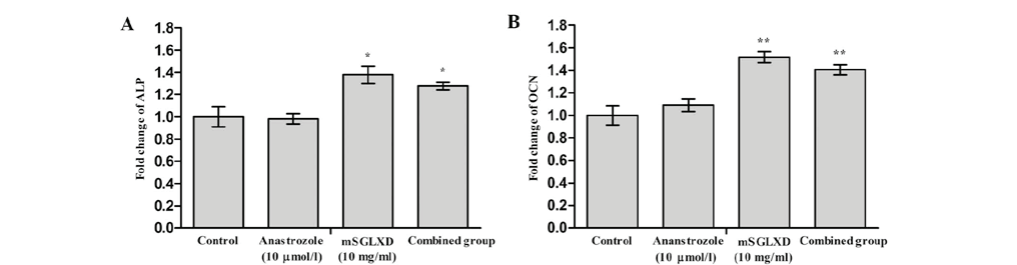
Effects of mSGLXD and anastrozole alone or in combination on ALP and OCN mRNA expression levels in MC3T3-E1 cells. Cells were treated with mSGLXD (10 mg/ml) and anastrozole (10 μmol/l) alone or in combination (combined group) for 48 h. (A) ALP and (B) OCN mRNA expression levels were determined by polymerase chain reaction. Values are expressed as the mean ± standard deviation of three independent experiments. ^*^P<0.05, ^**^P<0.01 vs. control group. ALP, alkaline phosphatase; OCN, osteocalcin; mSGLXD, modified Shu-Gan-Liang-Xue decoction; mRNA, messenger RNA.

Based on the results of the aforementioned experiments, 10 μmol/l anastrozole and 10 mg/ml mSGLXD were used for the following experiments. The PCR analysis results indicated that ALP and OCN mRNA expression levels were increased following treatment with mSGLXD alone or combined with anastrozole, in comparison with those of the control group (P<0.05 and P<0.01, respectively; Fig. 4). No significant change was observed in the anastrozole only treatment group.

### mSGLXD alone or in combination with anastrozole enhances ALP activity and OCN protein expression

**Figure 5:**
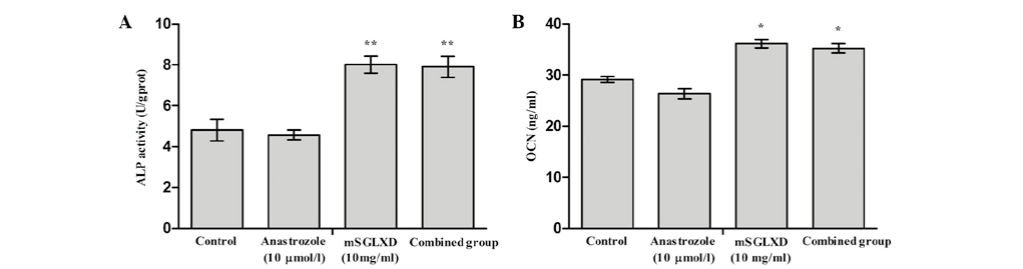
Effects of mSGLXD and anastrozole alone or in combination on ALP activity and OCN content of MC3T3-E1 cells. Cells were treated with mSGLXD (10 mg/ml) and anastrozole (10 μmol/l) alone or in combination (combined group) for 48 h. (A) ALP activity was determined using an ALP kit. ^**^P<0.01 vs. control group. (B) OCN concentration was determined by ELISA, ^*^P<0.05 vs. control group. Values are expressed as the mean ± standard deviation. ALP, alkaline phosphatase; OCN, osteocalcin; mSGLXD, modified Shu-Gan-Liang-Xue decoction; gprot, grams of protein.

The effects of mSGLXD and anastrozole alone or in combination on ALP activity and OCN content in MC3T3-E1 cells are exhibited in Fig. 5A and B. In the presence of 10 mg/ml mSGLXD alone or combined with 10 μmol/l anastrozole, ALP activity and OCN content were significantly increased following 48 h of culture (P<0.01 and P<0.05, respectively), while 10 μmol/l anastrozole had no significant effect.

### mSGLXD alone or in combination with anastrozole enhances bone mineralization of MC3T3-E1 cells

**Figure 6:**
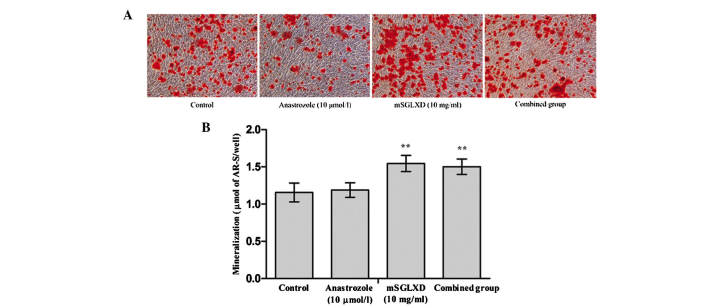
Mineralization nodules of MC3T3-E1 cells treated with mSGLXD and anastrozole alone or in combination. MC3T3-E1 cells were cultured in differentiation medium (α-MEM supplemented with 10% FBS, 10 mmol/l β-glycerophosphate and 50 μg/ml ascorbic acid) with mSGLXD (10 mg/ml) and anastrozole (10 μmol/l) alone or in combination (combined group) for 21 days. The mineralization nodules were visualized with AR-S staining. (A) Digital images (magnification, ×100) and (B) quantification values of AR-S staining. Values are presented as the mean ± standard deviation. ^**^P<0.01 vs. control group. mSGLXD, modified Shu-Gan-Liang-Xue Decoction; AR-S, alizarin red S.

AR-S staining is a standard method used for the visualization of nodular patterns and calcium deposition in MC3T3-E1 cell cultures *in vitro*. As shown in Fig. 6A, following culture in differential medium supplemented with β-glycerophosphate and ascorbic acid for 21 days, treatment with 10 mg/ml mSGLXD alone or in combination with anastrozole markedly increased AR-S staining in MC3T3-E1 cells. The AR-S concentration in the mSGLXD and combined groups demonstrated significant differences compared with that of the control group (P<0.01, Fig. 6B). Concurrent with the results of the other experiments, 10 μmol/l anastrozole did not influence the mineralization of MC3T3-E1 cells.

## Discussion

Phytoestrogens, natural estrogen-like substances contained in plant food, demonstrated estrogenic activities through binding to the ER and exhibiting ER-mediated estrogenic properties (37). Epidemiological and experimental data regarding the association between phytoestrogens and breast cancer risk or progression are inconsistent (37–40). Therefore, the safety of phytoestrogens for patients with breast cancer has remained to be elucidated. Clinically, Rhizoma Drynariae is added to mSGLXD to tonify the kidneys and strengthen the bones, while Caulis Piperis Kadsurae and Caulis Trachelospermi are supplemented as collateral-dredging and pain-relieving herbs. According to the results of a previous study, RD may possess estrogenic activity (32); however, no study had demonstrated that Caulis Piperis Kadsurae and Caulis Trachelospermi were phytoestrogens. Therefore, in the present study, the estrogenic activities of mSGLXD and RD were evaluated by dual-luciferase reporter assay-based bioluminescent measurements. In accordance with previously reported results, the results of the present study confirmed that RD had certain estrogenic properties and that low concentrations of RD stimulated MCF-7 cell proliferation (32,33). Of note, despite the addition of phytoestrogen RD, mSGLXD was found to not possess estrogenic activity. Furthermore, mSGLXD significantly inhibited MCF-7 cell proliferation following supplementation of the original drug SGLXD with RD and two additional herbs, and the removal of Radix Cynanchum Strati and Fructus Schisandrae. A possible explanation may be that since mSGLXD is a Traditional Chinese Medicine composed of numerous herbs, the estrogenic activity of RD may be modulated or counteracted by the presence of other bioactive components, which may have an antagonistic effect on the ER signaling pathway. Therefore, the results indicated that mSGLXD was safe for patients with breast cancer and also had certain anti-tumor effects on breast cancer cells.

Formation of new bone is the task of osteoblasts; therefore, enhancing osteoblast proliferation and differentiation is a potential therapeutic strategy for bone loss. During the formation phase of the bone cycle, ALP is expressed in markedly high quantities and therefore becomes an indicator of bone formation activity and a useful clinical therapeutic monitoring index (41). OCN, another classical biomarker of osteoblast cell function, was also investigated (41,42). The results of the present study indicated that mSGLXD not only stimulated MC3T3-E1 cell proliferation, but also upregulated ALP and OCN gene and protein expression levels. High concentrations of anastrozole markedly inhibited MC3T3-E1 cell proliferation. However, this inhibitory effect of anastrozole on MC3T3-E1 cell growth was alleviated by the addition of mSGLXD. Furthermore, mSGLXD (10 mg/ml) increased the mineralization of MC3T3-E1 cells induced by β-glycerophosphate and ascorbic acid. These results indicated that mSGLXD had anabolic effects on bone via the promotion of osteoblastic proliferation and differentiation, suggesting that it may provide a useful pathway for the prevention and treatment of AIBL.

Aromatase, an enzyme of the cytochrome P-450 superfamily and the product of the CYP19 gene, catalyzes the aromatization of C19 steroids (androstendione, testosterone and 16α-hydroxyandrostendione) to E_1_ and E_2_ (43). Aromatase is also the target of AIs, which are widely used in breast cancer endocrine therapy at present. Previous research by our group indicated that SGLXD, the original form of mSGLXD, simultaneously downregulated aromatase and steroid sulfatase at transcription and protein levels in ER-positive breast cancer cell lines MCF-7 and T47D (44), which may underlie the anti-tumor mechanism of SGLXD. Therefore, SGLXD may have synergistic inhibitory effects on aromatase with AIs to a certain extent. However, to elucidate whether the mSGLXD used in the present study exhibits a similar dual inhibitory effect on aromatase and steroid sulfatase requires further investigation. Bone is dynamically balanced by bone formation and bone resorption (45); therefore, the effects of mSGLXD on osteoclasts requires further investigation and conclusions should be confirmed by studies *in vivo*.

In conclusion, the present study demonstrated that mSGLXD not only inhibited breast cancer cell proliferation but also stimulated osteoblastic cell proliferation and differentiation. Furthermore, the inhibitory effect of anastrozole on osteoblastic cell growth was abrogated in the presence of mSGLXD. These results suggested that mSGLXD was a promising adjuvant therapy with high safety and efficacy in the prevention and treatment of AIBL in patients with breast cancer receiving AI treatment.

## Figures and Tables

**Table I:** Components of Shu-Gan-Liang-Xue decoction (SGLXD) and modified SGLXD (mSGLXD).

Chinese name	English name	Botanical name	mSGLXD (g)	SGLXD (g)
Baiwei	Radix Cynanchum Strati	*Cynanchum atratum Bunge*, in *Asclepiadaceae*	-	15
Mudanpi	Tree peony bark	*Paeonia suffruticosa* Andr.	15	15
Baishao	White peony root	*Paeonia lactiflora* Pall.	15	15
Chaihu	Chinese thorowax root	*Bupleurum chinense* DC.	10	10
Yujin	Wenchow turmeric root tuber	*Curcuma aromatic* Salisb.	10	10
Wuweizi	Fructus Schisandrae	*Schisandra chinensis* (Turcz.) Baill.	-	15
Gusuibu	Rhizoma Drynariae	*Drynaria fortunei* (Kunze ex Mett.) J. Sm.	15	-
Haifengteng	Caulis Piperis Kadsurae	*Piper kadsura* (Choisy) Ohwi.	15	-
Luoshiteng	Caulis Trachelospermi	*Trachelospermum jasminoides* (Lindl.) Lem.	15	-

**Table II:** Primers for polymerase chain reaction.

Target gene	Primers (5′-3′)	Annealing temperature	Amplification length (bp)
Alkaline phosphatase	TCCTGACCAAAAACCTCAAAGG TGCTTCATGCAGAGCCTGC	60°C	101
Osteocalcin	CTCACAGATGCCAAGCCCA CCAAGGTAGCGCCGGAGTCT	60°C	98
GAPDH	GGTGAAGGTCGGTGTGAACG CTCGCTCCTGGAAGATGGTG	62°C	233
